# In Vitro and In Vivo Evaluation of *Lactobacillus delbrueckii* subsp. *bulgaricus* KLDS1.0207 for the Alleviative Effect on Lead Toxicity

**DOI:** 10.3390/nu9080845

**Published:** 2017-08-08

**Authors:** Bailiang Li, Da Jin, Shangfu Yu, Smith Etareri Evivie, Zafarullah Muhammad, Guicheng Huo, Fei Liu

**Affiliations:** 1 Key Laboratory of Dairy Science, Ministry of Education, Northeast Agricultural University, Harbin 150030, China; 15846092362@163.com (B.L.); 18846420263@163.com (D.J.); yushangf@163.com (S.Y.); besta_intercom@yahoo.com (S.E.E.); zaf_wahla@hotmail.com (Z.M.); 2 Food Science and Nutrition Unit, Department of Animal Science, Faculty of Agriculture, University of Benin, PMB 1154 Benin City, Nigeria; 3 Food College, Northeast Agricultural University, Harbin 150030, China

**Keywords:** *Lactobacillus delbrueckii* subsp. *bulgaricus*, lead toxicity, adsorption, antioxidative activity

## Abstract

Lead (Pb) is a toxic contaminating heavy metal that can cause a variety of hazardous effects to both humans and animals. In the present study, *Lactobacillus delbrueckii* subsp. *bulgaricus* KLDS1.0207 (*L. bulgaricus* KLDS1.0207), which has a remarkable Pb binding capacity and Pb tolerance, was selected for further study. It was observed that the thermodynamic and kinetic model of *L. bulgaricus* KLDS1.0207 Pb binding respectively fit with the Langmuir–Freundlich model and the pseudo second-order kinetic model. Scanning electron microscopy and energy dispersive spectroscopy analysis disclosed that the cell surfaces were covered with Pb and that carbon and oxygen elements were chiefly involved in Pb binding. Combined with Fourier transform infrared spectroscopy analysis, it was revealed that the carboxyl, phosphoryl, hydroxyl, amino and amide groups were the main functional groups involved in the Pb adsorption. The protective effects of *L. bulgaricus* KLDS1.0207 against acute Pb toxicity in mice was evaluated by prevention and therapy groups, the results in vivo showed that *L. bulgaricus* KLDS1.0207 treatment could reduce mortality rates, effectively increase Pb levels in the feces, alleviate tissue Pb enrichment, improve the antioxidant index in the liver and kidney, and relieve renal pathological damage. Our findings show that *L. bulgaricus* KLDS1.0207 can be used as a potential probiotic against acute Pb toxicity.

## 1. Introduction

Lead (Pb) is a toxic contaminating heavy metal, with no constructive biological role, that remains a public health concern. The key sources of Pb in the human environment are diet, cosmetics, Pb-based paint, soil and dust from Pb-contaminated paint, gasoline, mining, and industrial activity [1,2,3]. Pb can be inhaled, as it can enter the atmosphere through industrial burning, smelting, and the emissions of vehicles with leaded gasoline. Pb can also enter drinking water through water supply pipes. Consumption of contaminated food and water are also potent sources of Pb exposure and toxicity. Pb is immensely toxic even at low concentrations, and mainly accumulates in bones, the brain [4,5], the liver, kidneys [6] and muscles causing several serious disorders, such as oxidative stress [6,7], carcinogenesis [8], disruption of calcium homeostasis, degenerative changes, nervous disorders [9,10], and sickness and tissue diseases, predominantly in children [11]. The half-life of Pb in blood plasma is 27 days, in blood is 35 days, in the brain it is about two years, and in bone it may persist for decades [12,13]. Oxidative stress is an imbalance between reactive oxygen species (ROS) and cellular antioxidant systems. Many studies propose oxidative stress as one of the significant mechanisms of the toxic effects of Pb [5,14,15].

The standard treatment for heavy metal poisoning is chelation therapy with the most commonly used chelating agents being CaNa_2_EDTA and Meso-2,3-dimercaptosuccinic acid (DMSA). However, adverse effects, including renal injury, malaise, nausea, vomiting, skin reactions [16,17] and progressive deficiencies of copper, zinc and other essential trace nutrients, which are an indispensable part of the body’s antioxidant defenses [18]. Therefore, nontoxic natural alternatives of chelating agents have been studied in recent years. 

It has been reported that some lactic acid bacteria (LAB) strains, including *L. rhamnosus*, *L. plantarum*, and *Leuconostoc mesenteroides,* are capable of binding and removing heavy metals, such as silver, cadmium and lead in vitro [19,20,21]. Furthermore, various recent studies have revealed that LAB can effectively protect against metal-induced oxidative stress through antioxidation in vivo [22,23,24]. Based on this, LAB may serve as a candidate to relieve the symptoms of heavy metal toxicity and their fermented products can be used as dietary fortification against heavy metal poisoning.

The aim of the present study was to select a novel probiotic LAB strain with high Pb-binding capacity and investigate different possible doses to treat acute Pb-induced tissue damage through biochemical enzyme analyses and histopathological studies. This study provides useful information on the utilization of LAB to aid recovery from Pb poisoning.

## 2. Materials and Methods

### 2.1. Chemicals and Reagents

Kits used to measure the levels of malondialdehyde (MDA; Njjcbio A003-1 kit), total superoxide dismutase (T-SOD; Njjcbio A001-1 kit), glutathione (GSH; Njjcbio A006-2kit), GSH peroxidase (GSH-PX; Njjcbio A005 kit), aspartate aminotransferase (AST; Njjcbio C010-2 kit), alanine aminotransferase (ALT; Njjcbio C009-2 kit) and a protein quantification kit, rapid (Njjcbio A045-2,) were procured from the Nanjing Jiancheng Bioengineering Institute (Nanjing, China). Lead dinitrate, lead acetate and other analytical reagents were purchased from the Tianli Chemical Reagent Company (Tianjin, China).

### 2.2. Bacterial Strain and Culture

*L. rhamnosus* KLDS1.0205, KLDS1.0911 and KLDS1.0912, *L. bulgaricus* KLDS1.0207 and KLDS1.9201, *L. plantarum* KLDS1.0386 and KLDS1.0344, *L. acidophilus* KLDS1.1003, *L. helveticus* KLDS1.0903 and *L. casei* KLDS1.0351 were isolated from traditional dairy products in Sinkiang Province, China and identified by API 50CH strips and 16S rRNA gene similarity analysis. These LAB strains were stored at the Key Laboratory of Dairy Science (KLDS), Ministry of Education, China. All strains were anaerobically incubated in De Man, Rogosa and Sharpe (MRS) broth (Hopebio Company, Qingdao, China) at 37 °C for 18 h and were sub-cultured twice prior to the experiment.

### 2.3. Estimation of Pb Binding and Pb Tolerance

The Pb binding ability of 10 strains was analyzed as previously described with little modification [25]. All strains were incubated for 18 h and the cultured biomass was centrifuged at 8000 rpm for 20 m. The centrifuged mass was washed twice with ultrapure water to obtain cell pellets. The bacterial concentration was adjusted to 1 g/L (wet weight) using ultrapure water containing 50 mg/L lead dinitrate and samples were then incubated at 37 °C for 24 h (pH 6.0). The samples were centrifuged at 8000 rpm for 20 m and the residual Pb concentrations of the supernatants were measured by flame atomic absorption spectrophotometry (Spectra AA 220; Varian, Palo Alto, CA, USA). The metal removal efficiency based on mass balance was calculated using the following equation:

$$\mathrm{Removal}(\%)=\frac{C_{i}-C_{e}}{C_{i}}\times 100\%$$

where, C_i_ and C_e_ are the initial Pb concentration and residual Pb concentration after removal, respectively.

The Pb tolerance of each strain was determined by the minimum inhibitory concentration (MIC) approach [26]. MRS agar medium containing 50 to 1000 mg/L lead dinitrate solution was prepared, and 10 μL of cultured LAB strain was spotted on the MRS agar medium at an inoculum level of 1 × 10^9^ CFU/mL. LAB growth was recorded after cultivation at 37 °C for 48 h. The minimum concentration of Pb that completely inhibited LAB growth was considered as the MIC in this study.

### 2.4. Equilibrium Isotherm and Kinetic Study

The equilibrium isotherm was performed as previously reported [27], and the harvested cell pellets were suspended in ultrapure water containing 5 to 75 mg/L lead dinitrate to give a final bacterial concentration of 1 g/L (dry weight). The Pb binding assay was then conducted with an initial pH of 6.0 and the equilibrium content of Pb bound by the bacterium was expressed as follows:

(1) $$q_{e}(\mathrm{mg}\;\mathrm{metal}/g\;\mathrm{biosorbent})=\frac{C_{i}-C_{e}}{m/V}$$

where, C_i_ and C_e_ are the initial Pb concentration and residual Pb concentration after removal, respectively; and m/V = 1 g/L.

The Langmuir, Freundlich and Langmuir–Freundlich models were used to determine the sorption equilibrium between the biosorbent and metal ions. Isotherm constants for the three models were obtained by non-linear regression methods [28,29].

The kinetic study was conducted as previously described [27], the harvested cell pellets were suspended in ultrapure water containing 50 mg/L lead dinitrate to give a final bacterial concentration of 1 g/L (dry weight). The Pb binding assay was then conducted with an initial pH of 6.0 and the concentration of Pb in the supernatant was detected at different time intervals up to 240 m.

The pseudo first and second-order rate equations were used in the kinetic model of Pb biosorption with the integrated form of the pseudo first-order model as follows:

$$\mathrm{lg}\;\frac{q_{e}}{q_{e}{-q}_{t}}=\frac{k_{1}}{2.303}t$$

where q_t_ is adsorption capacity; and time t and k_1_ are first-order rate constants. k_1_ can be determined from the slope of the plot of $\mathrm{lg}\frac{q_{e}}{q_{e}{-q}_{t}}$ vs. t.

The pseudo second-order integrate equation is expressed as:

$$\frac{t}{q_{t}}=\frac{1}{k_{2}q_{e}^{2}}+\frac{1}{q_{e}}t$$

where k_2_ can be determined from the interception of the linearized plot of t/q_t_ vs. t.

### 2.5. Scanning Electron Microscopy (SEM) Analysis

The samples for SEM observation were prepared as described earlier [30]. The untreated harvested cell pellets and those treated with Pb (50 mg/L) were fixed by 2.5% glutaraldehyde (*v*/*v*) at 4 °C for 1.5 h, and washed thrice with phosphate buffer solution. Supernatants were discarded and the cell pellets treated with different concentrations (50%, 70%, 90% and 100%) of alcohol as well as a mixture of alcohol and t-butanol (1:1) to wash cells successively. Finally, the cell pellets were eluted with plain t-butanol. The specimens were then put in a freeze-drier for 4 h and sputter-coated with gold. The scanning and photography were performed using SEM with an energy dispersive spectrometer (EDS).

### 2.6. Fourier Transform Infrared Spectroscopy (FT-IR) Analysis

The untreated harvested cell pellets and those treated with Pb (50 mg/L) were lyophilized and mixed with KBr powder as KBr discs. Discs containing 2% (*w*/*w*) of finely ground powder of each sample were prepared. The FT-IR technique was employed to characterize the changes in the functional groups on the untreated cell pellets and those treated with Pb.

### 2.7. In Vivo Protective Potential of L. bulgaricus KLDS1.0207 Against Acute Pb Toxicity

#### 2.7.1. Animals and Experimental Design

A total of 72 female BALB/c mice (6–7 weeks old, weighing 16 to 20 g) were purchased from the Vital River Laboratory Animal Technology Company (Beijing, China) and housed in a room under controlled environmental conditions at 25 °C and with a 12-h light/dark cycle. All the mice were put into plastic cages for one week before the begin of the experiments. The experimental protocol was approved by the Institutional Animal Care and Use Committee of the Northeast Agricultural University under the approved protocol number Specific pathogen free rodent management (SRM)-06.

Mice were fed with standard commercial pellets and water ad libitum and were randomly divided into two major groups. The prevention and therapy groups were divided into three subgroups and six subgroups, respectively. Each subgroup had a total of eight BALB/c mice. The details of the administration are shown in Table 1. On the basis of Azar’s studies [31], the oral dose of lead acetate [(CH_3_COO)_2_Pb·3H_2_O] was 100 mg/kg body weight. The dose of DMSA used in this study was 50 mg/kg/day [17,32].

All of the therapy groups had their feces collected every week to determine Pb concentration. The animals were then sacrificed under ether anesthesia, and blood samples were collected with Pb-free needles, and all tissue samples were obtained and kept at −80 °C for biochemical assays and estimation of the Pb concentration. Some kidney samples were put into 10% neutral formalin to analyze their pathology.

#### 2.7.2. Determination of Pb in Blood, Feces and Tissue

Samples were digested in concentrated HNO_3_ by using a microwave digestion system. Pb concentration in the livers, kidneys, blood, and feces was determined by a graphite furnace atomic absorption spectrophotometer.

#### 2.7.3. Biochemical Assays

The levels of MDA and GSH and the activities of T-SOD and GSH-Px in the mouse kidneys and livers, and the activities of ALT and AST in the mouse serum were measured by using the assay kit in accordance with the recommendations of the manufacturer.

#### 2.7.4. Histopathological Studies

The kidneys were fixed in 10% neutral formalin for 48 h. Samples were then embedded in paraffin, sliced into 5 μm thickness and stained with hematoxylin-eosin (H&E) for examination by light microscopy.

### 2.8. Statistical Analysis

All values are expressed as the mean ± standard deviation (SD). A minimum of three independent experiments were carried out for each assay. The statistical significance of data comparisons was determined using one-way analysis of variance (ANOVA), followed by Duncan’s multiple range test. Values of *p* < 0.05 were considered to be statistically significant.

## 3. Results 

### 3.1. Pb Biosorption and Pb Tolerance of LAB Strains

**Figure 1:**
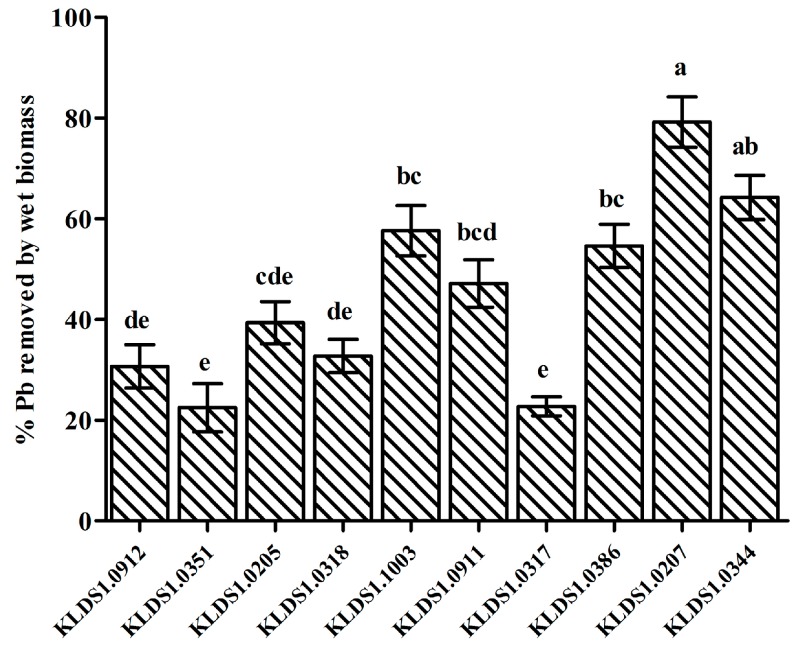
The Pb-binding ability of the tested lactic acid bacteria (LAB) strains when incubated with an initial Pb concentration of 50 mg/L. Values are mean ± SD of three determinations. Significant differences (*p* < 0.05) among the strains are indicated with different letters above the graphical bars. KLDS1.0205, KLDS1.0911 and KLDS1.0912 are *L. rhamnosus*. KLDS1.0207 and KLDS1.9201 are *L. bulgaricus*. KLDS1.0386 and KLDS1.0344 are *L. plantarum*. KLDS1.1003 is *L. acidophilus*. KLDS1.0903 is *L. helveticus*. KLDS1.0351 is *L. casei*.

The Pb-binding abilities of the 10 strains are presented in Figure 1. While the initial Pb concentration was 50 mg/L, the range of Pb removal by the tested strains was from 22.47% to 79.18%. *L. bulgaricus* KLDS1.0207 showed the best binding ability among the 10 strains. Moreover, *L. bulgaricus* KLDS1.0207 had the highest MIC value (>1000 mg/L) (Table 2). Based on these characteristics, *L. bulgaricus* KLDS1.0207 was selected as a candidate for the further study of its biosorption mechanism and in vivo assays.

### 3.2. Biosorption Isotherms and Kinetic Models

**Figure 2:**
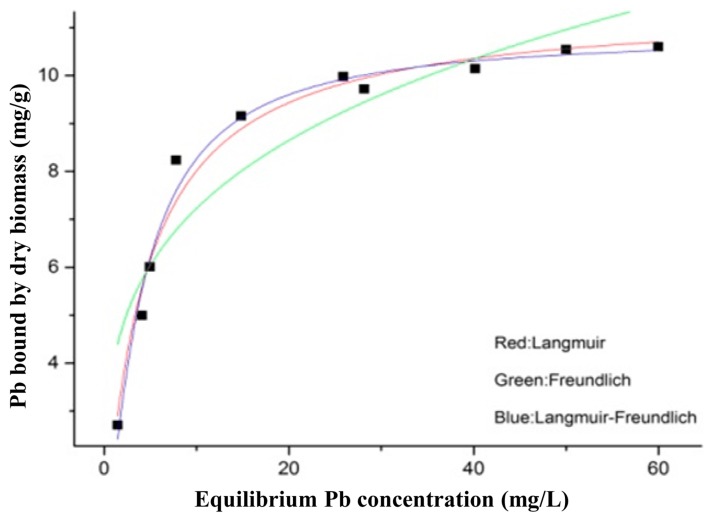
Adsorption isotherm of Pb binding by *L. bulgaricus* KLDS1.0207.

As indicated in Figure 2, the Pb concentration in the solution was positively correlated with the Pb-binding ability of *L. bulgaricus* KLDS1.0207. The data of different isotherm models, including Langmuir, Freundnlich and Langmuir–Freundlich, are listed in Table S1, respectively. The values of the coefficients of correlation (*R*^2^) illustrated that the Langmuir–Freundlich model (*R*^2^ = 0.9820) best suited our experimental data.

**Figure 3:**
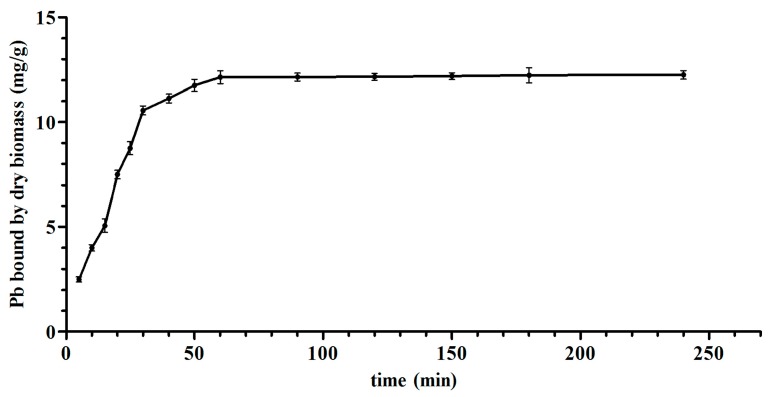
Pb binding of *L. bulgaricus* KLDS1.0207 at different time points. Values are mean ± standard deviation (SD) of three determinations.

As shown in Figure 3, the biosorption of Pb onto *L. bulgaricus* KLDS1.0207 was efficient, because the binding process was nearly completed in 60 m. The rate kinetics of the reaction adopted pseudo first-order kinetic model (*R*^2^ = 0.9665) for first 15 m (Figure S1A). Further analysis showed that the pseudo second-order kinetic model (*R*^2^ = 0.9916) was far better at explaining the biosorption of Pb on the surface of *L. bulgaricus* KLDS1.0207 for the complete process (Figure S1B).

### 3.3. Electron Microscopy Analysis

**Figure 4:**
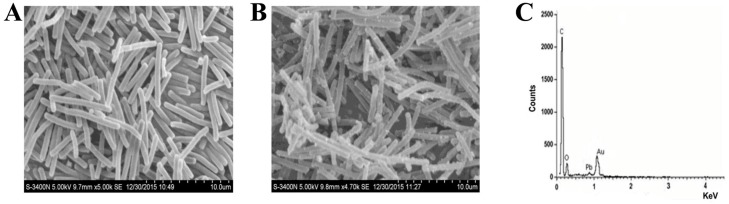
Scanning electron micrographs of *L. bulgaricus* KLDS1.0207 untreated and treated with Pb (50 mg/L): (**A**) Untreated biomass; (**B**) Biomass after lead binding; and (**C**) Energy dispersive spectrometer (EDS) analysis of biomass after Pb binding. Scale bar = 10.0 μm.

The SEM micrographs of *L. bulgaricus* KLDS1.0207 after the treatment with Pb indicated that many light clusters of metal precipitates were localized but did not cover the cell surface evenly (Figure 4B). However, no light precipitates were found on the surface of untreated cell pellets (Figure 4A). The EDS analysis confirmed that the presence of Pb resulted in the light precipitates (Figure 4C, Table S2).

### 3.4. FT-IR Analysis

**Figure 5:**
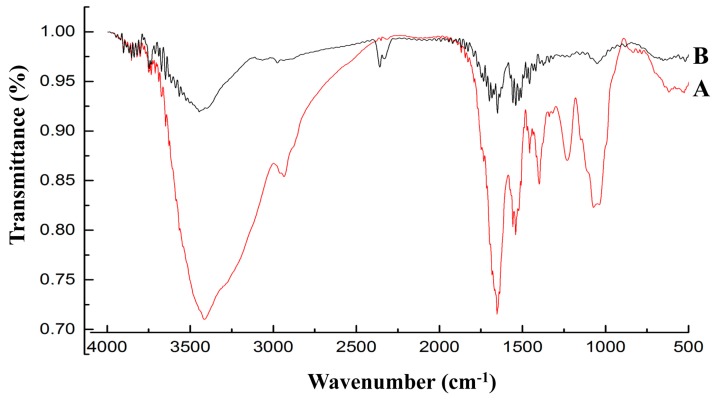
Fourier transform infrared spectrums of *L. bulgaricus* KLDS1.0207 untreated and treated with Pb (50 mg/L): (**A**) Untreated biomass; and (**B**) Biomass after Pb binding.

The FT-IR spectrums of untreated *L. bulgaricus* KLDS1.0207 and that treated with Pb (50 mg/L) are shown in Figure 5. The marked shift to a strong wave number at 3500–3200 cm^−1^ may be due to the interaction of the –NH group of amide and −OH group of alcohol-phenol with Pb. In the 1720–1700 cm^−1^ region, a C=O stretching vibration of carboxylic acid was observed. The amine II band (1400–1410 cm^−1^) is related to a combination of the NH in-plane bending mode with the stretching of the C−N peptide bond. The disappearance of the peak at 1250–1150 cm^−1^ indicated that the P=O and O−H or C−O stretching vibrations of polysaccharides could play a role in Pb biosorption. These constituents can be combined with Pb by the main functional carboxyl, phosphoryl, hydroxyl, amino, and amide groups.

### 3.5. Mortality and Viscera Index Analysis

The survival rates of BALB/c mice are presented in Table 3. No mortality was recorded in the pre-control group and the mortality of pre-LAB group (1/8) was lower than the pre-Pb group (2/8). The mortality in the low dose and medial dose group were 1/8, no death was recorded in the high dose and drug group, indicating that the high dose group played a role in mitigating Pb toxicity.

In the viscera indices, the pre-Pb group was significantly different (*p* < 0.05) from the pre-control group, suggesting that some pathological changes may be present in the pre-Pb group. There was no significant difference between the pre-LAB group and the pre-control group, which may be due in part to the presence of *L. bulgaricus* KLDS1.0207. The high dose and Pb group were significantly different in the liver body ratio (*p* < 0.05), but no significant difference in the kidney body ratio was found between the high dose and control group. This was dissimilar from the low dose, medial dose and drug group, which may imply that the high dose group had a better protection against Pb exposure than the drug group as the long-term intake DMSA may have resulted in some damage to the liver and kidneys.

### 3.6. Pb Levels in Feces and Tissues

The changes in the Pb levels in the feces of the mice in the therapy groups are presented in Table 4. In the first week, in comparison to the Pb group, the fecal Pb levels of all treated groups increased significantly (*p* < 0.05). No dose–response relationship by the administration of *L. bulgaricus* KLDS1.0207 was observed. During the second week, the excretion amounts of Pb in the Pb group and treated groups were lower than the first week, indicating that *L. bulgaricus* KLDS1.0207 and DMSA were effective against acute Pb toxicity in the first week.

The Pb concentrations of the blood, liver and kidneys in the pre-LAB group were significantly lower than the pre-Pb group (*p* < 0.05). In the blood and kidneys, the decreasing Pb levels in all dose groups were significantly different from the values obtained for the Pb group (*p* < 0.05), whereas no significant differences were observed in the liver. This implies that *L. bulgaricus* KLDS1.0207 is more efficient in the kidney than in the liver (Table 5).

### 3.7. Activity of Antioxidant Enzymes

**Figure 6:**
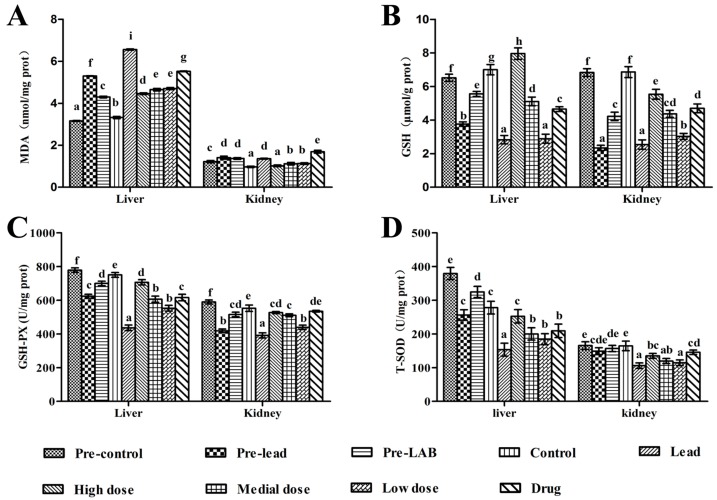
Effects of *L. bulgaricus* KLDS1.0207 on Pb-induced variations of antioxidant capacity in the liver and kidney of mice: (**A**): Malondialdehyde (MDA); (**B**): Glutathione (GSH); (**C**): Glutathione peroxidase (GSH-PX); and (**D**): Total superoxide dismutase (T-SOD). Values are mean ± SD. Significant differences (*p* < 0.05) among the groups are indicated with different letters above the graphical bars.

The levels of antioxidant capacity in the liver and kidney of mice are shown in Figure 6. In both prevention and therapy groups, acute Pb exposure induced a remarkable increase in the levels of MDA and a marked decrease in the levels of GSH, GSH-PX and T-SOD in the liver. All *L. bulgaricus* KLDS1.0207 administrations had significant protective effects on the antioxidant capacity (*p* < 0.05). Particularly, the high dose group was significantly different with the low dose, medial dose and drug group, indicating that the high dose of *L. bulgaricus* KLDS1.0207 could more efficiently relieve oxidative stress in the liver. Generally, in the kidney, there were significant differences in MDA, GSH and GSH-PX levels in all dose group (*p* < 0.05), however, a significant increase in T-SOD levels was only observed in the high dose group. The MDA levels of the pre-LAB group showed non-significant decrease trends compared with the pre-Pb group. There was a decreasing pattern in the MDA level of all dose groups (*p* < 0.05). In contrast, the drug group was significantly higher than the Pb group in the MDA level (*p* < 0.05), indicating that the drug group had a significantly adverse effect on the kidney. In all, the high dose of *L. bulgaricus* KLDS1.0207 could serve as an effective antioxidant in the liver and kidney.

The activities of marker enzymes in the serum of mice are shown in Table 6. Although no significant difference in the activity of ALT was observed in the prevention group, a significant decrease (*p* < 0.05) in the AST/ALT ratio between the pre-LAB group and the pre-Pb group was noticed. In the therapy group, the AST/ALT ratio in all treated groups was significantly reduced (*p* < 0.05), especially the high dose group showed the lowest AST/ALT ratio.

### 3.8. Histopathology

**Figure 7:**
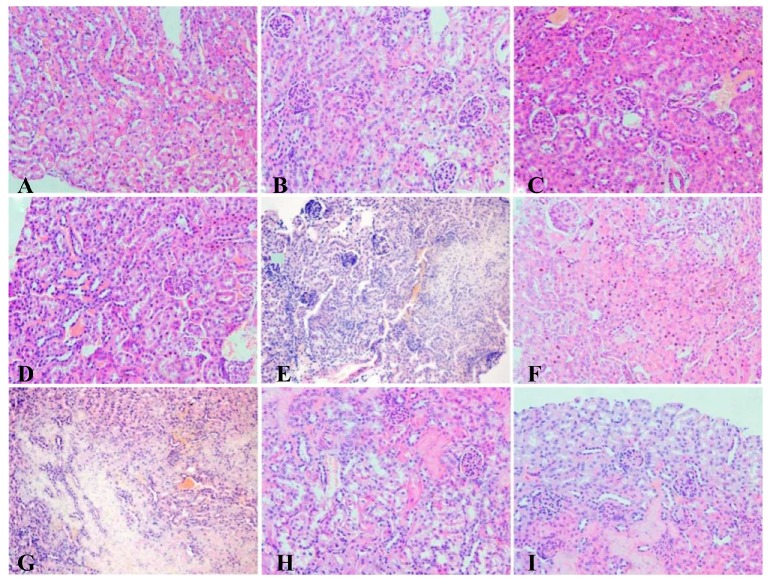
Representative photomicrographs of renal tissue of mice (hematoxylin-eosin (H&E) staining; magnification ×400): (**A**) Normal appearance of the renal tissue of mice in the control prevention group; (**B**) Renal tissue of mice in the pre-lead group; (**C**) Renal tissue of mice in the pre-LAB group; (**A**) Renal tissue of mice in the control therapy group; (**B**) Renal tissue of mice in the lead-only therapy group; (**C**) Renal tissue of mice in the high-dose therapy group; (**D**) Renal tissue of mice in the medial-dose group; (**E**) Renal tissue of mice in the low-dose group; and (**F**) Renal tissue of mice in the drug group.

In the control groups (Figure 7A,D), normal kidney histomorphology was apparent. In the pre-Pb group (Figure 7B), glomeruli were hyperemic, the glomerular volume became significantly bigger and few glomeruli were missed, some renal tubular epithelial cells showed swelling, which was alleviated in the pre-LAB group (Figure 7C). The symptoms of all treated groups (Figure 7F–I) including inflammatory cells, swelled tubular epithelial cells and granular degeneration and glomerular hyperemic were ameliorated to some extent when compared to the Pb group (Figure 7E).

## 4. Discussion

Pb is a prevalent environmental pollutant with no beneficial biological role. It adversely induces oxidative stress damage to the host. Many researches have reported that LAB can not only sequester Pb, but also relieve the oxidative damage [1,2]. Screening LAB strains against Pb toxicity should take a number of properties into consideration. First, LAB strains must show high Pb-binding ability, enabling them to bind Pb before the intestinal absorption of Pb by the host. Second, LAB strains should exhibit high resistance to Pb to avoid it being poisoned. Furthermore, to perform Pb removal in the gastrointestinal tract, it is necessary for the screened strains to remain viable in high concentrations of bile and stomach acids. In the present study, *L. bulgaricus* KLDS1.0207 showed the best binding aptitude among 10 strains (Figure 1) and had a remarkable tolerance to Pb (Table 2). In addition, *L. bulgaricus* KLDS1.0207 showed good resistance to simulated gastrointestinal tract conditions (data not shown). Based on these properties, *L. bulgaricus* KLDS1.0207 was selected as a potential strain for the further study of its Pb biosorption mechanism and in vivo assays.

Various mechanisms of metal biosorption, including adsorption, ion exchange, complexation, chelation and microprecipitation have been proposed because of differences in the bacterial structures among the species [33]. The cell walls of the gram-positive bacteria consist of peptidoglycans, teichoic acids, proteins and polysaccharides [34,35]. These contents also contain negatively charged functional groups, which serve as the primary sites of metal ion sorption on a bacterium surface [36]. The phosphate and carboxyl group present in peptidoglycans and teichoic acids are the primary sites of metal ion binding on the surface of the bacterial cell [37]. SEM micrographs and EDS analysis (Figure 4) confirmed that the added toxic Pb metal was localized on the cell surface evenly. A previous study has reported a similar phenomenon in *L. mesenteroides* after Pb binding [21], this phenomenon may be related to the passive physicochemical adsorption mechanism.

By analyzing the FT-IR spectrum (Figure 5), it was able to be speculated that the functional groups (carboxyl, phosphoryl, hydroxyl, amino and amide) of biological macromolecules (fatty acids, polysaccharides, S-layer proteins and teichoic acid) bind Pb through complexation, ion exchange and physical adsorption (electrostatic attraction). These predictions were consistent with the previous assertion that the carboxyl, hydroxyl and amide groups were involved in Pb uptake [38,39,40]. It may be necessary in future studies to elucidate the effects of the specific groups in this mechanism.

The thermodynamic and kinetic models of *L. bulgaricus* KLDS1.0207 binding Pb fit with the Langmuir–Freundlich model and pseudo second-order kinetic model, respectively (Figure 2 and Figure S1), this is in line with the cadmium binding characterization of an acidophilic bacterium [27], indicating that adsorption (physical and chemical) is a complex process. 

As *L. bulgaricus* KLDS1.0207 had an excellent Pb-binding capacity, the protective effects of it against acute Pb toxicity in mice was evaluated using prevention and therapy groups. In the present study, mice were orally given a lead acetate solution of 100 mg/kg bodyweight to stimulate acute Pb toxicity, followed by treatment with different concentrations of *L. bulgaricus* KLDS1.0207 for two weeks. Our study showed that the high dose therapy group had a higher ability to increase fecal excretion of Pb than low dose groups (Table 4). Similar results were observed for the other *Lactobacillus* strains, which could modulate intestinal heavy metal absorption in mice by increasing fecal heavy metal excretion [22,23,41].

The high concentration of Pb in different tissues was associated with increased oxidative reaction, which might be responsible, at least in part, for Pb-induced toxic effects [42]. Several studies have reported a possible link between oxidative stress and the disruption of metal ion homeostasis [43,44,45]. Our results showed that *L. bulgaricus* KLDS1.0207 lowered Pb-induced oxidative stress and facilitated a protective role in reducing the lipid peroxide by decreasing MDA concentration and improving other antioxidants, such as T-SOD, GSH and GSH-Px in the liver and kidneys (Figure 6).

Changes in the ALT and AST levels are often used for liver pathological examination, and AST/ALT is an important biochemical indicator [46,47]. ALT mainly exists in the liver cell plasma. When liver cell damage is lower, changes in liver-cell-membrane permeability elevate ALT levels in the blood. On the other hand, increased AST blood levels are a result of severely damaged liver cells. It thus follows that higher AST/ALT ratios imply more severe liver cell damage. The treatment of *L. bulgaricus* KLDS1.0207 could improve the ALT and AST levels (Table 6). 

Compared with the dose groups, the drug group was able to increase Pb excretion. Nonetheless, the drug DMSA has been shown in a previous study to have some adverse effects, including depletion of zinc and copper in the body [17]. In all, the results from the present study have shown that the most effective dose in the therapy groups was 1 × 10^10^ CFU/mL of *L. bulgaricus* KLDS1.0207 in 0.4 mL skim milk. This was similar to the findings of earlier studies that 2 × 10^10^ CFU/mL of *L. plantarum* CCFM8246 (0.2 mL) and 1 × 10^9^ Colony-Forming Units (CFU) of *L*. *plantarum* CCFM8610 (0.5 mL) were effective against copper and cadmium toxicity in mice, respectively [22,41]. These effective LAB strains can be freeze-dried or spray-dried to obtain the strain powders for the practical administration. Interestingly, LAB strains such as *L. plantarum* and *L. rhamnosus* can improve the absorption and bioavailability of several trace elements in animals [48,49]. Thus, it is very significant to exploit LAB as a heavy metal removal agent added to food or feed.

## 5. Conclusions

*L. bulgaricus* KLDS1.0207 had high Pb biosorption and Pb tolerance in vitro. The adsorption process of Pb was complex and efficient by the main functional groups, including the carboxyl, phosphoryl, hydroxyl, amino and amide groups. *L. bulgaricus* KLDS1.0207 facilitated Pb detoxication in vivo by increasing Pb levels in the feces, alleviated tissue Pb enrichment, improved the antioxidant index, and relieved renal pathological damages. Therefore, *L. bulgaricus* KLDS1.0207 may be used as a novel probiotic candidate against acute Pb toxicity.

## Figures and Tables

**Table 1:** Experimental design of the prevention and therapy groups.

Groups	Treatment on the Indicated Day(s) for the Following:	
Prevention groups	1–14 days	15 day
Pre-control (*n* = 8)	SM	PW
Pre-Pb (*n* = 8)	SM	Pb
Pre-LAB (*n* = 8)	SM + LAB	Pb
Therapy groups	1 day	2–15 days
Control (*n* = 8)	PW	SM
Pb (*n* = 8)	Pb	SM
High dose (*n* = 8)	Pb	SM + LAB
Medial dose (*n* = 8)	Pb	SM + LAB
Low dose (*n* = 8)	Pb	SM + LAB
Drug (*n* = 8)	Pb	DMSA(50 mg/kg/day)

PW, 0.4 mL plain water. SM, 0.4 mL skim milk. Pb, [(CH_3_COO)_2_Pb·3H_2_O] at 2 mg in 0.4 mL plain water. SM + LAB, in the Pre-LAB group, 1 × 10^9^ CFU/mL *L. bulgaricus* KLDS1.0207 in 0.4 mL skim milk. SM + LAB in the high, medial and low dose group, 1 × 10^10^, 1 × 10^9^ and 1 × 10^8^ CFU/mL *L. bulgaricus* KLDS1.0207 in 0.4 mL skim milk, respectively. Meso-2,3-dimercaptosuccinic acid (DMSA), DMSA at 1 g in 0.4 mL 5% sodium bicarbonate solution. All modes of administration were oral.

**Table 2:** Pb tolerance of the tested LAB strains.

Strains	Minimum Inhibitory Concentration for Pb (mg/L)
*L*. *bulgaricus* KLDS1.0207	>1000
*L*. *bulgaricus* KLDS1.9201	200
*L*. *helveticus* KLDS1.0903	350
*L*. *acidophilus* KLDS1.1003	300
*L*. *plantarum* KLDS1.0386	150
*L*. *plantarum* KLDS1.0344	450
*L*. *rhamnosus* KLDS1.0205	100
*L*. *rhamnosus* KLDS1.0911	50
*L*. *rhamnosus* KLDS1.0912	400
*L*. *casei* KLDS1.0351	200

**Table 3:** The mortality and the viscera indices of different groups.

Groups	Mortality	Liver Body Ratio	Kidney Body Ratio
Pre-control	0/8	3.80 ± 0.25 ^b^	1.17 ± 0.03 ^bc^
Pre-Pb	2/8	3.28 ± 0.22 ^a^	1.06 ± 0.04 ^a^
Pre-LAB	1/8	3.65 ± 0.23 ^ab^	1.16 ± 0.05 ^bc^
Control	0/8	4.95 ± 0.31 ^d^	1.42 ± 0.06 ^d^
Pb	2/8	3.33 ± 0.14 ^a^	1.11 ± 0.04 ^ab^
High dose	0/8	4.47 ± 0.27 ^c^	1.40 ± 0.03 ^d^
Medial dose	1/8	3.43 ± 0.19 ^ab^	1.31 ± 0.03 ^c^
Low dose	1/8	3.37 ± 0.28 ^ab^	1.21 ± 0.04 ^c^
Drug	0/8	3.39 ± 0.15 ^ab^	1.23 ± 0.07 ^c^

Values are mean ± SD. Significant differences (*p* < 0.05) among different groups are indicated with different superscript letters.

**Table 4:** Effects of *L. bulgaricus* KLDS1.0207 on Pb level in feces under different time.

Groups	Pb (μg/g)	
	First Week	Second Week
Control	0.16 ± 0.03 ^a^	0.13 ± 0.04 ^a^
Pb	23.15 ± 1.35 ^b^	0.40 ± 0.13 ^b^
High dose	31.15 ± 2.32 ^c^	0.65 ± 0.14 ^bc^
Medial dose	29.48 ± 1.12 ^c^	0.52 ± 0.10 ^bc^
Low dose	28.81 ± 1.87 ^c^	0.41 ± 0.18 ^b^
Drug	42.81 ± 2.43 ^d^	0.70 ± 0.16 ^c^

Values are mean ± SD. Significant differences (*p* < 0.05) among different groups are indicated with different superscript letters.

**Table 5:** Effects of *L. bulgaricus* KLDS1.0207 on Pb levels in the blood and tissue of mice.

Groups	Blood (μg/L)	Liver (μg/g)	Kidney (μg/g)
Pre-control	0.32 ± 0.02 ^a^	0.15 ± 0.02 ^a^	0.17 ± 0.03 ^a^
Pre-Pb	383.30 ± 23.12 ^f^	1.51 ± 0.04 ^e^	1.72 ± 0.07 ^g^
Pre-LAB	344.03 ± 24.32 ^e^	1.13 ± 0.09 ^d^	1.25 ± 0.06 ^f^
Control	0.43 ± 0.05 ^a^	0.12 ± 0.04 ^a^	0.16 ± 0.04 ^a^
Pb	302.20 ± 25.32 ^d^	0.46 ± 0.08 ^c^	0.66 ± 0.08 ^e^
High dose	234.12 ± 10.18 ^c^	0.35 ± 0.07 ^bc^	0.45 ± 0.06 ^bc^
Medial dose	248.01 ± 7.54 ^c^	0.39 ± 0.05 ^c^	0.54 ± 0.04 ^cd^
Low dose	258.33 ± 8.13 ^c^	0.43 ± 0.07 ^c^	0.63 ± 0.05 ^de^
Drug	176.14 ± 6.17 ^b^	0.27 ± 0.06 ^b^	0.36 ± 0.08 ^b^

Values are mean ± SD. Significant differences (*p* < 0.05) among different groups are indicated with different superscript letters.

**Table 6:** Effects of *L. bulgaricus* KLDS1.0207 on Pb-induced variations in the activity of marker enzymes in the serum of mice.

Groups	ALT (U/L)	AST (U/L)	AST/ALT
Pre-control	33.56 ± 2.13 ^a^	55.87 ± 3.01 ^a^	1.67 ± 0.02 ^a^
Pre-Pb	40.74 ± 1.76 ^c^	84.23 ± 3.32 ^c^	2.07 ± 0.01 ^b^
Pre-LAB	38.67 ± 2.38 ^bc^	65.89 ± 4.21 ^b^	1.70 ± 0.00 ^a^
Control	35.39 ± 3.01 ^ab^	100.23 ± 4.54 ^d^	2.84 ± 0.11 ^c^
Pb	46.19 ± 2.88 ^d^	228.12 ± 5.65 ^h^	4.95 ± 0.19 ^g^
High dose	34.23 ± 1.47 ^a^	123.33 ± 4.25 ^e^	3.60 ± 0.03 ^d^
Medial dose	37.87 ± 2.13 ^abc^	153.33 ± 4.02 ^f^	4.05 ± 0.12 ^e^
Low dose	40.02 ± 2.42 ^c^	167.88 ± 5.23 ^g^	4.20 ± 0.12 ^ef^
Drug	33.87 ± 2.12 ^a^	146.46 ± 4.37 ^f^	4.33 ± 0.14 ^f^

Values are mean ± SD. Significant differences (*p* < 0.05) among different groups are indicated with different superscript letters. AST, aspartate aminotransferase; and ALT, alanine aminotransferase.

## Supplementary Materials

The following are available online at www.mdpi.com/2072-6643/9/8/845/s1, Figure S1: Kinetic models of the Pb binding by *L. bulgaricus* KLDS1.0207. (A) Pseudo first-order kinetic model of the Pb binding by *L. bulgaricus* KLDS1.0207 in the first 15 m. (B) Pseudo second-order kinetic model of the Pb binding by *L. bulgaricus* KLDS1.0207. Table S1: Adsorption constants derived from simulations with different isotherm models. Table S2: Quantitative analysis of EDS.
