# Supplementary material for: In Vitro and In Vivo Evaluation of Lactobacillus delbrueckii subsp. bulgaricus KLDS1.0207 for the Alleviative Effect on Lead Toxicity
Source: Nutrients. 2017 Aug 8;9(8):845. doi: 10.3390/nu9080845 (PMC5579638; doi:10.3390/nu9080845)
Supplement: Supplementary file 1 [file nutrients-09-00845-s001.zip › nutrients-212111-supplementary.pdf]

Table S1. Adsorption constants derived from simulations with different isotherm models.

Name of isotherm model	Constants	
1、Langmuir	Qm	15.47
$q_e = \frac{q_m \times b \times C_e}{1 + b \times C_e}$	b	0.2494
	R^2	0.9731
2、Freundlich	K_F	3.9836
$q_e = K_F \times C_e^{1/n}$	N	3.8658
	R^2	0.8574
3、Langmuir-Freundlich	K_{LF}	1.9337
$q_e = \frac{K_{LF} \times C_{eq}^{1/n}}{1 + a C_{eq}^{1/n}}$	A	0.1778
	N	1.2500
	R^2	0.9820

q_e (mg metal/g biosorbent) represents the equilibrium content of the Pb bound by the biomass. C_e (mg/L) represents the equilibrium Pb concentration.

Table S2. Quantitative analysis of EDS.

Element	Weight (%)	Atomic (%)
C	66.22	85.43
O	13.39	12.97
Au	18.95	1.49
Pb	1.45	0.11

C, carbon; O, oxygen; Au, gold; and Pb, lead.

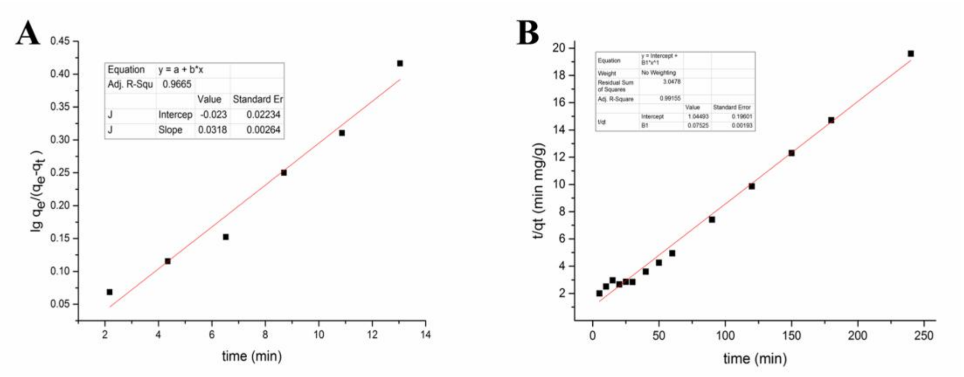

Figure S1. Kinetic models of the Pb binding by *L. bulgaricus* KLDS1.0207: (**A**) Pseudo first-order kinetic model of the Pb binding by *L. bulgaricus* KLDS1.0207 in the first 15 min; and (**B**) Pseudo second-order kinetic model of the Pb binding by *L. bulgaricus* KLDS1.0207.
